# Refractory hyponatremia in small cell lung cancer: a case report and literature review

**DOI:** 10.3389/fendo.2026.1780594

**Published:** 2026-03-11

**Authors:** Suqing Bao, Ling Qin, Yang Zhang, Xia Jiang, Lijun Duan

**Affiliations:** Department of Endocrinology, Tianjin First Central Hospital, Tianjin, China

**Keywords:** diabetes, hyponatremia, small cell lung cancer, syndrome of inappropriate antidiuretic hormone secretion, tolvaptan

## Abstract

The clinical manifestations of lung cancer are diverse. Presentation initially as the Syndrome of Inappropriate Antidiuretic Hormone Secretion (SIADH) is relatively uncommon. This article reports the diagnosis and treatment process of a 76-year-old male patient with hyponatremia, ultimately diagnosed with SIADH attributed to small cell lung cancer (SCLC). The patient presented with fatigue and significant hyponatremia. Comprehensive investigation excluded adrenal, thyroid, and other functional abnormalities. Combined with features including high urinary sodium, euvolemic status, and normal renal function, the findings were consistent with a diagnosis of SIADH. Subsequent imaging and pathological examinations revealed the etiology to be SCLC. Management included fluid restriction, sodium supplementation, and the use of tolvaptan. Subsequent chemotherapy and radiotherapy for the SCLC led to the gradual normalization of serum sodium. This article reviews the literature in conjunction with this case, summarizing the diagnostic approach to hyponatremia, as well as the diagnostic workup, pathophysiological mechanisms, and treatment strategies for SIADH, aiming to enhance the early recognition and comprehensive management of this condition.

## 1 Clinical data

A 76-year-old male was admitted in June 2025 due to nausea, poor appetite, and fatigue for one month.

History of Present Illness: In May 2025, the patient experienced unexplained poor appetite, nausea, and fatigue, with low spirits. There was no significant cough, sputum, abdominal pain, diarrhea, or fever. The symptoms were initially neglected. Two days prior to admission, the fatigue worsened, accompanied by poor appetite, nausea, and intermittent vomiting of gastric contents (non-projectile). He presented to our emergency department. Laboratory tests revealed severe hyponatremia, hypokalemia, and hypochloremia, with mild hyperglycemia. Following intravenous sodium and potassium supplementation, serum electrolytes showed minimal improvement, and there was no significant relief of nausea, anorexia, or fatigue. The patient experienced an unintentional weight loss of approximately 5 kg over the past month.

Personal History: Denied smoking and alcohol use.

Past Medical History: Hypertension for more than 10 years, currently taking nifedipine controlled-release tablets 30 mg once daily, with well-controlled blood pressure. No prior history of diabetes mellitus or hyperglycemia.

Family History, Marital and Obstetric History: Unremarkable.

Physical Examination: T 36.5 °C, P 78 bpm, R 18 bpm, BP 130/90 mmHg; alert and well-developed. No dryness of lips or skin; no edema of the lower extremities.

Laboratory Investigations:

At the emergency department: serum Na 114.5 mmol/L↓, K 3.27 mmol/L↓, Cl 76.7 mmol/L↓, and Glucose (GLU) 8.01 mmol/L↑.

On admission: Serum Na 120.2 mmol/L↓, K 2.90 mmol/L↓, Cl 84.2 mmol/L↓, Ca 2.01 mmol/L↓, P 0.83 mmol/L↓; Serum Osmolality: 259.17 mOsm/kg H_2_O↓; Urine pH: 7.5, Specific Gravity 1.017, Urine osmolality: 456 mOsm/kg H_2_O; Arterial Blood Gas: PH 7.487, Standard Base Excess (SBE) 0.7, Actual Base Excess (ABE) 1.3; Adrenocorticotropic Hormone (ACTH) (8 am) 17.405 pg/mL; Serum Cortisol (8 am) 16.393 µg/dL, (4 pm) 12.742 µg/dL, (0 am) 14.325 µg/dL; 24h Urinary Cortisol 377.64 µg/24h; 24h Urinary VMA 2.32 mg/24h; 24h Urine Volume ~2700 mL, Urine Na 118.7 mmol/L, Urine Cl 108.3 mmol/L, Urine Protein 0.32 g/24h, Urine K 16.86 mmol/L, Urine Ca 2.07 mmol/L, Urine P 3.7 mmol/L; Thyroid Function: Free Triiodothyronine (FT_3_) 3.68 pmol/L↓ (Ref 3.85-6.30), Free Thyroxine (FT_4_) 18.90 pmol/L, Thyroid-Stimulating Hormone (TSH) 0.63 mIU/L↓ (Ref 0.75-5.60); Parathyroid Hormone (PTH) 77.8 pg/mL, Calcitonin 0.637 pg/mL; Pituitary and Gonadal Hormones: Prolactin (PRL), Testosterone (TESTO), Luteinizing Hormone (LH), Estradiol (E2), Follicle-Stimulating Hormone (FSH), Progesterone (PROG), Growth Hormone (GH) all within normal limits; Renin-Angiotensin-Aldosterone System (RAAS): (Supine) Plasma Renin 1.704 pg/mL↓ (2.4-32.8), Angiotensin II 67.438 pg/mL, Aldosterone 86.481 pg/mL (10-160), Aldosterone-to-Renin Ratio (ARR): 50.75; (Upright) Renin 3.349 pg/mL↓ (3.8-38.8), Angiotensin II 91.422 pg/mL, Aldosterone 124.111 pg/mL (40-310), ARR: 37.06; After serum potassium normalization, Captopril Suppression Test: (Upright 1h) Serum Cortisol: 32.404 µg/dL, Plasma Renin 9.406 pg/mL, Aldosterone 192.165 pg/mL, ARR: 20.43; (1h post-dose) Serum Cortisol: 43.170 µg/dL, Renin 24.395 pg/mL, Aldosterone 174.490 pg/mL, ARR: 7.15; (2h post-dose) Serum Cortisol: 31.278 µg/dL, Renin 25.403 pg/mL, Aldosterone 164.490 pg/mL, ARR:6.48; Hemoglobin A1c (HbA1c): 9.7%; Oral Glucose Tolerance Test (OGTT) + Insulin Release Test: Fasting Glucose 5.58 mmol/L, 1 hour (1h) Glucose 15.58 mmol/L, 2h Glucose 17.85 mmol/L↑(Ref <7.8); Fasting Insulin 14.70 µIU/mL, 1h Insulin 68.30 µIU/mL, 2h Insulin 103.00 µIU/mL; Tumor Markers: Carcinoembryonic Antigen (CEA) 7.59 ng/mL↑ (0-5.0), Carbohydrate Antigen 19-9 (CA199) 46.4 U/mL↑ (Ref 0-30), Pro-Gastrin-Releasing Peptide (Pro-GRP): 94.9 pg/mL↑ (Ref 0-69.2); Alpha-Fetoprotein (AFP), Carbohydrate Antigen 724 (CA724), Neuron-Specific Enolase (NSE), Cytokeratin 19 Fragment (CYFRA 21-1), Carbohydrate Antigen 125 (CA125), Carbohydrate Antigen 153 (CA153) were unremarkable. Biochemistry: Serum β_2_-microglobulin: 1.66 mg/L, Uric Acid 84.4 µmol/L↓ (Ref 208-428), Albumin (ALB): 39.9 g/L↓ (Ref 40-55), Triglyceride (TG) 0.63 mmol/L, Low-Density Lipoprotein Cholesterol (LDL-C): 2.03 mmol/L.

Ancillary Investigations: Echocardiography: Basal septal hypertrophy. Adrenal CT: Bilateral adrenal gland thickening, irregular renal contours. Thyroid Ultrasound: Multiple nodules in both thyroid lobes (TI-RADS 3). Pituitary MRI: No abnormalities. Brain MRI + DWI: Bilateral frontal subdural effusions, white matter hyperintensities (Fazekas grade 1), cerebral atrophy. Chest CT: Nodule in the left upper lobe, PET recommended; multiple micronodules in both lungs. Chest Contrast-enhanced CT: Solid nodular shadow in the left upper lobe, enlarged lymph node in the right lower paratracheal region of the mediastinum, suggestive of left upper lobe malignancy with mediastinal lymph node metastasis; multiple solid and ground-glass micronodules in both lungs, suggestive of bronchitis; fullness of hilar and some mediastinal lymph nodes. Positron Emission Tomography/Computed Tomography (PET/CT): Near round soft tissue density nodule near the left upper lobe hilum, approximately 1.4 cm × 1.4 cm, with abnormal tracer uptake, SUV_max_ 21.2. Multiple reticular, patchy, and ground-glass opacities in both lungs, with heterogeneous tracer uptake, SUV 1.4. Enlarged lymph node in the pretracheal space, size 2.4 cm × 1.7 cm, with abnormal tracer uptake, Standardized Uptake Value (SUV) 6.4. Bilateral pleural mild thickening, no abnormal tracer uptake. Pathological examination of transbronchial needle aspiration (TBNA) from the subcarinal lymph node region: Neuroendocrine tumor, favoring small cell carcinoma; Immunohistochemistry: Tumor cells positive for Creatine Kinase (CK), Chromogranin A (CgA) (partial weak), Synaptophysin (Syn), Insulinoma-associated protein 1 (INSM1), and Cluster of Differentiation 56 (CD56); negative for Thyroid Transcription Factor-1 (TTF-1), Napsin A, and P40; Leukocyte Common Antigen (LCA) positive in lymphocytes; Antigen Kiel 67 (Ki-67) index hot spot approximately 70%.

## 2 Treatment course

Based on the clinical presentation, laboratory, and ancillary findings, euvolemic hyponatremia was considered. Concurrent high urinary sodium (118.7 mmol/L), normal adrenal cortical hormones, thyroid function, pituitary hormones, and RAAS levels after potassium correction, absence of diuretic use or history of renal insufficiency, along with elevated tumor markers and a suspicious lung nodule, strongly suggested malignancy-associated SIADH as the initial diagnosis.

Additionally, hypokalemia present during the course was investigated. Common causes such as thyroid disorders, cortisol abnormalities, immune diseases, and renal tubular acidosis were actively excluded. Considering recent poor intake, potassium supplementation was initiated, and serum potassium normalized. The Captopril Suppression Test performed after potassium correction showed significant suppression of aldosterone and a marked decrease in the ARR ratio, essentially ruling out primary aldosteronism as the cause of hypokalemia.

Notably, the patient had comorbid type 2 diabetes. According to recent international consensus/expert opinions and related studies (1–4), for SIADH patients with refractory chronic fluid restriction who also have type 2 diabetes, Sodium-Glucose Linked Transporter 2 (SGLT2) inhibitors can be considered. By promoting the excretion of osmotic substances (glucose) and free water, they can effectively raise serum sodium levels. Therefore, in addition to conventional intravenous hypertonic saline, this case was treated with the SGLT2 inhibitor dapagliflozin for glycemic control, which was achieved satisfactorily.

**Figure 1:**
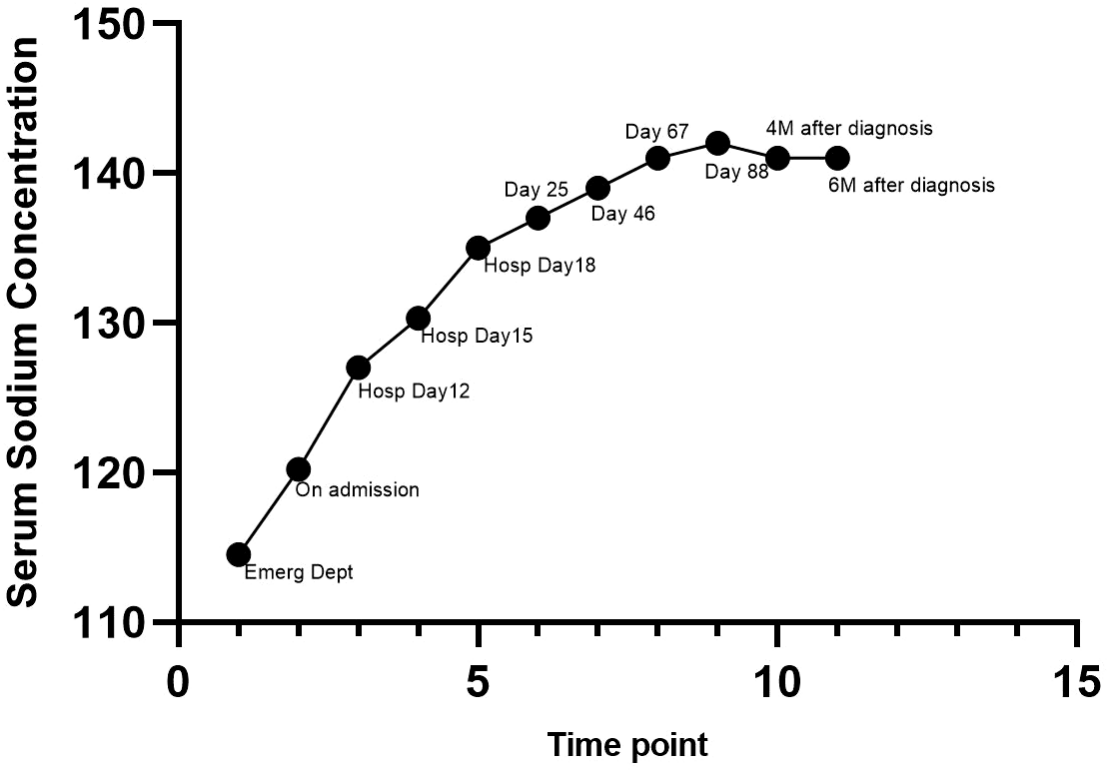
Changes in Serum Sodium Concentration During the Whole Treatment Period. 1. At the emergency department: Presented with severe hyponatremia (Na: 114.5 mmol/L); 2. On admission: Initial management with fluid restriction, intravenous sodium supplementation, and oral salt capsules (6 g daily), with limited efficacy (Na: 120.2–124 mmol/L); 3. Day 12 of hospitalization: Oral salt capsules (6 g/d) were continued, and tolvaptan was initiated and gradually titrated from 7.5 mg/d to 37.5 mg/d. Serum sodium improved gradually (Na: 119–127 mmol/L); 4. Day 15 of hospitalization: Oral salt capsules (6 g/d), tolvaptan (37.5 mg/d), and SGLT2i were administered. Serum sodium improved gradually (Na: 130.3 mmol/L); 5. Day 18 of hospitalization: Oral salt capsules (6 g daily), tolvaptan (52.5 mg/d), and SGLT2i were continued. Serum sodium returned to the normal range for the first time (Na: 135 mmol/L); 6. Day 25: Transferred to the oncology department and received the first cycle of chemotherapy (etoposide plus carboplatin), combined with thoracic radiotherapy and hyperthermia as a radiosensitizing strategy. Oral salt capsules (6 g daily), tolvaptan (45 mg/d), and SGLT2i were continued (Na: 137 mmol/L); 7. Day 46: Received the second cycle of chemotherapy (etoposide plus carboplatin), with continuation of thoracic radiotherapy and hyperthermia for radiosensitization. The dosage of tolvaptan was tapered to 30 mg once daily (Na: 139 mmol/L); 8. Day 67: Received the third cycle of chemotherapy (etoposide plus carboplatin), with continuation of thoracic radiotherapy and hyperthermia for radiosensitization. The dosage of tolvaptan was tapered to 22.5 mg once daily (Na: 141 mmol/L); 9. Day 88: Received the fourth cycle of chemotherapy (etoposide plus carboplatin), with continuation of thoracic radiotherapy and hyperthermia for radiosensitization. Tolvaptan dose was reduced to 15 mg daily (Na: 142 mmol/L); 10. 4 months after diagnosis: Repeated imaging showed tumor regression, and serum sodium remained within normal limits. Tolvaptan dose was reduced to 7.5 mg daily with improved tumor control (Na: 136–141 mmol/L); 11. 6 months after diagnosis: Sustained tumor response was observed; serum sodium remained stable within the normal range. Tolvaptan dose was maintained at 7.5 mg daily (Na: 137–141 mmol/L).

During treatment, monitoring revealed limited efficacy of oral salt capsules at 6 g daily, intravenous sodium supplementation, and fluid restriction in increasing serum sodium levels. Given the high suspicion of SIADH, tolvaptan was initiated empirically for active correction of hyponatremia. Starting at 7.5 mg/day (half tablet), the dose was gradually titrated up to 37.5 mg/day (3.5 tablets) based on serum sodium monitoring. Serum sodium gradually increased from a nadir of 114.5 mmol/L to 130.3 mmol/L. Concurrently, the patient’s nausea and fatigue improved, and appetite largely recovered, indicating treatment efficacy. Following administration of tolvaptan (52.5 mg/d, 4.5 tablets), serum sodium returned to the normal range for the first time (Na: 135 mmol/L) (**Table 1**, **Figure 1**).

**Table 1:** Timeline of treatment response and serum sodium recovery.

Time point	Event/clinical status
At the emergency department	Presented with severe hyponatremia (Na: 114.5 mmol/L)
On admission	Initial management with fluid restriction, intravenous sodium supplementation, and oral salt capsules (6 g daily), with limited efficacy (Na: 120.2–124 mmol/L)
Day 12 of hospitalization	SIADH was highly suspected. Oral salt capsules (6 g/d) were continued, and tolvaptan was initiated and gradually titrated from 7.5 mg/d to 37.5 mg/d. Serum sodium improved gradually (Na: 119–127 mmol/L)
Day 15 of hospitalization	Oral salt capsules (6 g/d), tolvaptan (37.5 mg/d), and SGLT2i were administered. Serum sodium improved gradually (Na: 130.3 mmol/L)
Day 18 of hospitalization	Oral salt capsules (6 g daily), tolvaptan (52.5 mg/d), and SGLT2i were continued. Serum sodium returned to the normal range for the first time (Na: 135 mmol/L)
Day 25	Transferred to the oncology department and received the first cycle of chemotherapy (etoposide plus carboplatin), combined with thoracic radiotherapy and hyperthermia as a radiosensitizing strategy. Oral salt capsules (6 g daily), tolvaptan (45 mg/d), and SGLT2i were continued (Na: 137 mmol/L)
Day 46	Received the second cycle of chemotherapy (etoposide plus carboplatin), with continuation of thoracic radiotherapy and hyperthermia for radiosensitization. The dosage of tolvaptan was tapered to 30 mg once daily (Na: 139 mmol/L)
Day 67	Received the third cycle of chemotherapy (etoposide plus carboplatin), with continuation of thoracic radiotherapy and hyperthermia for radiosensitization. The dosage of tolvaptan was tapered to 22.5 mg once daily (Na: 141 mmol/L)
Day 88	Received the fourth cycle of chemotherapy (etoposide plus carboplatin), with continuation of thoracic radiotherapy and hyperthermia for radiosensitization. Tolvaptan dose was reduced to 15 mg daily (Na: 142 mmol/L)
4 months after diagnosis	Repeated imaging showed tumor regression, and serum sodium remained within normal limits. Tolvaptan dose was reduced to 7.5 mg daily with improved tumor control (Na: 136–141 mmol/L)
6 months after diagnosis	Sustained tumor response was observed; serum sodium remained stable within the normal range. Tolvaptan dose was maintained at 7.5 mg daily (Na: 137–141 mmol/L)

Subsequently, efforts focused on identifying the etiology of SIADH. Guided by the suspicious lung lesion, further chest contrast-enhanced CT and whole-body PET/CT indicated a metabolically active left upper lobe nodule with mediastinal lymph node metastasis. To obtain a pathological diagnosis, bronchoscopy-guided biopsy of the suspicious lymph node was performed, confirming a neuroendocrine tumor. Immunohistochemistry supported small cell lung cancer (SCLC). The patient was ultimately diagnosed with SIADH secondary to SCLC. After diagnosis, the patient was transferred to oncology and received 21−day cycles of etoposide plus carboplatin chemotherapy, combined with thoracic radiotherapy and hyperthermia as a radiosensitizing strategy. Serum sodium levels remained acceptable during treatment. As the tumor regressed and the disease was controlled, the tolvaptan dose was gradually reduced to 15 mg/day (1 tablet). Serum sodium stabilized between 137–142 mmol/L (**Table 1**, **Figure 1**).

## 3 Follow-up

After discharge, regular outpatient follow-up was conducted for 6 months. During the follow-up period, serum sodium levels remained stably within the normal range (136–141 mmol/L) without recurrence of hyponatremia, and no obvious adverse reactions related to medications were observed. Regarding tumor treatment response, reexamination of chest contrast-enhanced CT and tumor markers at the 4th and 6th months after discharge showed continuous tumor regression, with no signs of local recurrence or distant metastasis, indicating that the chemotherapy regimen achieved satisfactory disease control. The patient’s general condition was good, with significant improvement in clinical symptoms such as fatigue and lethargy, and the quality of life was effectively improved.

As shown in **Table 1**, the patient’s clinical course was characterized by a clear temporal relationship between tumor treatment, serum sodium correction, and medication adjustment. Following the confirmation of SCLC, sequential treatment including chemotherapy, radiotherapy, and hyperthermia was administered in a timely manner. Meanwhile, tolvaptan was carefully titrated from 7.5 mg to 52.5 mg daily according to the serum sodium level and tumor response, which allowed for effective and stable correction of hyponatremia. Subsequently, the tolvaptan dose was tapered to 7.5 mg once daily as the tumor responded to treatment.With continuous tumor control during follow-up, serum sodium remained within the normal range (137–141 mmol/L) without recurrence, indicating a favorable long-term therapeutic response.

## 4 Discussion

Hyponatremia (serum sodium < 135 mmol/L) is one of the most common electrolyte disorders. Epidemiological data demonstrate that its prevalence can reach as high as 35% in hospitalized patients and exceeds 40% in elderly inpatients, with approximately 25%–40% of cases attributed to SIADH (5). Even mild hyponatremia is significantly associated with adverse clinical outcomes, including prolonged hospital stay, increased readmission rates, higher healthcare resource consumption, and elevated all-cause mortality, which underscores its substantial clinical significance (5, 6).

The diagnosis of SIADH necessitates a systematic approach. First, non-hypotonic hyponatremia (e.g., that caused by hyperglycemia) must be excluded, followed by assessment of the patient’s volume status. Key laboratory criteria for diagnosis include hypotonic hyponatremia in a euvolemic state, accompanied by inappropriately elevated urine osmolality (typically > 100 mOsm/kg) and increased urinary sodium excretion (> 30 mmol/L), while ruling out other confounding factors such as adrenal insufficiency, hypothyroidism, renal failure, and recent diuretic use (7, 8). This patient’s clinical presentation fulfilled these classic criteria: euvolemic hypotonic hyponatremia, high urinary sodium (118.7 mmol/L), no history of diuretic use or renal insufficiency, and no significant abnormalities in endocrine hormones, including adrenal corticosteroids, thyroid function, pituitary hormones, and the renin-angiotensin-aldosterone system (RAAS). For etiology screening, guidelines recommend prioritizing head and chest imaging when there are no clear drug-related causes, as pulmonary and intracranial pathologies are common secondary etiologies of SIADH (7). The diagnostic trajectory of this case was consistent with this recommendation, ultimately identifying an occult SCLC through chest contrast-enhanced CT, PET/CT, and bronchoscopic lymph node biopsy.

The etiologies of SIADH are diverse. Malignancy is a significant cause, with SCLC, due to its neuroendocrine properties, being the most frequently reported tumor associated with SIADH, likely through ectopic secretion of Arginine Vasopressin (AVP) or AVP-like substances by tumor cells (9, 10). Additionally, certain drugs (e.g., SSRIs, carbamazepine), pulmonary infections, and central nervous system disorders can induce SIADH (9). Notably, in elderly patients, some cases initially labeled “idiopathic” SIADH may reveal an underlying malignancy upon long-term follow-up, necessiting vigilance and periodic review (9).

Pathophysiologically, the core of SIADH lies in the “inappropriate” sustained secretion or enhanced activity of AVP, disengaging from normal plasma osmolality and volume regulation. This leads to increased water reabsorption in the renal collecting ducts, causing water retention, decreased plasma osmolality, and thus dilutional hyponatremia. Concurrent mild volume expansion suppresses the RAAS and promotes atrial natriuretic peptide release, collectively leading to increased urinary sodium excretion, manifesting as the characteristic “high urinary sodium” (11, 12). Extracellular hypotonicity causes water movement into cells, leading to cerebral edema, which is the pathological basis for neurological symptoms like fatigue, lethargy, confusion, and even seizures.

Furthermore, the pathophysiological mechanism underlying SIADH associated with SCLC has been well documented (13, 14). As a typical neuroendocrine tumor, SCLC cells possess the ability to ectopically synthesize and release AVP autonomously, independent of physiological feedback regulation by serum osmolality and body fluid volume. The unregulated and excessive AVP secreted by tumor cells binds to V2 receptors in the renal collecting ducts, upregulates the expression and translocation of aquaporin-2 channels, and markedly enhances free−water reabsorption—an effect that amplifies the core pathophysiological changes of SIADH described above. This paraneoplastic phenomenon is closely related to the neuroendocrine differentiation characteristics and abnormal gene expression profiles of SCLC. Given that hyponatremia may precede typical radiological and clinical manifestations of lung cancer, recognition of the pathophysiological link between SCLC and SIADH is critical for early etiological screening and timely intervention (13, 15, 16).

Regarding treatment, fluid restriction (typically <800–1000 mL/day) is the first-line foundational therapy for SIADH, aimed at creating a negative water balance (7, 17). Etiological treatment is fundamental. In this case, controlling the SCLC with chemotherapy led to resolution of the SIADH. Pharmacologically, vasopressin V2 receptor antagonists (e.g., tolvaptan) competitively block AVP action in the renal collecting ducts, effectively promoting water excretion without increasing sodium loss, thereby correcting hyponatremia. Multiple randomized controlled trials and clinical studies have confirmed their efficacy and safety (18, 19). They should be initiated at low doses with close monitoring of serum sodium to prevent overly rapid correction.

Conventional management of SIADH mainly relies on fluid restriction, hypertonic saline, vasopressin receptor antagonists, and urea, yet each has limitations such as poor tolerability, insufficient efficacy, or high cost. In recent years, SGLT2 inhibitors have emerged as a promising novel therapeutic strategy for SIADH, acting via glycosuria-induced osmotic diuresis to enhance electrolyte-free water excretion (20, 21). A 2023 prospective randomized crossover trial confirmed that SGLT2 inhibitors significantly elevated serum sodium concentrations and improved neurocognitive function in patients with chronic SIADH (3, 22). As an off-label, mechanism-driven, and increasingly recognized emerging approach, SGLT2 inhibitors offer a valuable alternative for refractory or comorbid cases. The application of dapagliflozin in this case represents an individualized, evidence-based practice, facilitating stable correction of hyponatremia and providing a clinically informative reference for the management of malignancy-associated SIADH complicated by type 2 diabetes.

This study has several inherent limitations that should be acknowledged when interpreting the findings. First, this is a single-case retrospective study, which inherently lacks statistical power due to the absence of a control group and a small sample size. Therefore, the conclusions drawn from this case cannot be generalized to all patients with malignancy-associated SIADH, and further prospective studies with larger cohorts are needed to validate the observations and inferences. Second, the patient had comorbid essential hypertension and type 2 diabetes mellitus, which are complex chronic conditions that may have interacted with the pathophysiological processes of SIADH and SCLC. These comorbidities could potentially confound the clinical manifestations, treatment responses, and outcomes, making it difficult to fully isolate the independent effects of SIADH and the underlying malignancy on the patient’s condition. Additionally, as a retrospective analysis, this study was subject to the limitations of existing medical records, including potential missing or incomplete data related to long-term follow-up, detailed medication adjustments, and subtle changes in laboratory parameters, which may have impacted the comprehensiveness of the analysis.

In summary, the diagnosis and management of hyponatremia should systematically exclude various etiologies. For SIADH as a common type, comprehensive judgment integrating clinical, biochemical, and imaging findings is required. Treatment should be individualized, combining fluid restriction, pharmacological therapy, and treatment of the underlying cause, aiming to rapidly stabilize serum sodium and improve prognosis.

## Data Availability

The original contributions presented in the study are included in the article/supplementary material. Further inquiries can be directed to the corresponding author.

## Glossary

ABE: Actual Base Excess
ACTH: Adrenocorticotropic Hormone
AFP: Alpha-Fetoprotein
ALB: Albumin
ARR: Aldosterone-to-Renin Ratio
AVP: Arginine Vasopressin
CA125: Carbohydrate Antigen 125
CA153: Carbohydrate Antigen 153
CA199: Carbohydrate Antigen 19-9
CA724: Carbohydrate Antigen 724
CD56: Cluster of Differentiation 56
CEA: Carcinoembryonic Antigen
CgA: Chromogranin A
CK: Creatine Kinase
CYFRA 21-1: Cytokeratin 19 Fragment
E2: Estradiol
FSH: Follicle-Stimulating Hormone
FT₃: Free Triiodothyronine
FT₄: Free Thyroxine
GLU: Glucose
GH: Growth Hormone
HbA1c: Hemoglobin A1c
INSM1: Insulinoma-Associated Protein 1
Ki-67: Antigen Kiel 67
LCA: Leukocyte Common Antigen
LDL-C: Low-Density Lipoprotein Cholesterol
LH: Luteinizing Hormone
NSE: Neuron-Specific Enolase
OGTT: Oral Glucose Tolerance Test
PET/CT: Positron Emission Tomography/Computed Tomography
PRL: Prolactin
PROG: Progesterone
Pro-GRP: Pro-Gastrin-Releasing Peptide
RAAS: Renin-Angiotensin-Aldosterone System
SBE: Standard Base Excess
SCLC: Small Cell Lung Cancer
SGLT2: Sodium-Glucose Cotransporter 2
SIADH: Syndrome of Inappropriate Antidiuretic Hormone Secretion
SUV: Standardized Uptake Value
Syn: Synaptophysin
TBNA: Transbronchial Needle Aspiration
TESTO: Testosterone
TSH: Thyroid-Stimulating Hormone
TG: Triglyceride
TTF-1: Thyroid Transcription Factor-1.
